# Prioritisation of food-borne parasites in Europe, 2016

**DOI:** 10.2807/1560-7917.ES.2018.23.9.17-00161

**Published:** 2018-03-01

**Authors:** Martijn Bouwknegt, Brecht Devleesschauwer, Heather Graham, Lucy J Robertson, Joke WB van der Giessen, the Euro-FBP workshop participants

**Affiliations:** 1 Centre for Infectious Disease Control Netherlands, National Institute for Public Health and the Environment, Bilthoven, the Netherlands; 2 Department of Public Health and Surveillance, Scientific Institute of Public Health (WIV-ISP), Brussels, Belgium; 3 Faculty of Veterinary Medicine, Utrecht University, Utrecht, the Netherlands; 4 Department of Food Safety and Infection Biology, Faculty of Veterinary Medicine, Norwegian University of Life Sciences, Oslo, Norway; 5 The Euro-FBP workshop participants are listed at the end of the article

**Keywords:** ranking, multi-criteria decision analysis, awareness, preparedness planning

## Abstract

Priority setting is a challenging task for public health professionals. To support health professionals with this and in following a recommendation from the Food and Agriculture Organization of the United Nations (FAO) and World Health Organization (WHO), 35 European parasitologists attended a workshop from 8–12 February 2016 to rank food-borne parasites (FBP) in terms of their importance for Europe and regions within Europe. **Methods:** Countries were divided into European regions according to those used by the European Society of Clinical Microbiology and Infectious Diseases. We used the same multicriteria decision analysis approach as the FAO/WHO, for comparison of results, and a modified version, for better regional representation. Twenty-five FBP were scored in subgroups, using predefined decision rules. **Results:** At the European level, *Echinococcus multilocularis* ranked first, followed by *Toxoplasma gondii* and *Trichinella spiralis*. At the regional level, *E. multilocularis* ranked highest in Northern and Eastern Europe, *E. granulosus* in South-Western and South-Eastern Europe, and *T. gondii* in Western Europe. Anisakidae, ranking 17th globally, appeared in each European region’s top 10. In contrast, *Taenia solium*, ranked highest globally but 10th for Europe. **Conclusions:** FBP of importance in Europe differ from those of importance globally, requiring targeted surveillance systems, intervention measures, and preparedness planning that differ across the world and across Europe.

## Introduction

Food-borne parasites (FBP) are increasingly recognised as a cause of health problems in humans [1-3]. The list of potential FBP contains 93 different species, differing in their public health relevance [4]. Well known examples of important FBP with a significant contribution to disease burden are *Toxoplasma gondii*, the cause of congenital toxoplasmosis following primary infection in pregnant women, and *Taenia solium*, the cause of neurocysticercosis [5]. Recently, the disease burden of 11 food-borne parasites was estimated to be 12 Disability Adjusted Life Years (DALY) per 100,000 population [3], which is nearly 900,000 DALY when extrapolated to the estimated European population of 740 million for 2015 according to the World Bank [6].

Cost-effective allocation of the limited resources available for reducing the public health burden associated with infectious diseases requires prioritisation. Various studies have focused on the identification and prioritisation of specific infectious diseases; for example, studies have quantified the global burden of food-borne diseases [5], ranked threats to livestock in the United Kingdom (UK) [7] and Australia [8], and ranked infectious threats to humans in the UK [9], Germany [10] and the Netherlands [11]. Ideally, such prioritisation studies are based on quantitative models and data that allow direct comparisons, as was done for quantifying the global burden of food-borne disease [5]. However, important aspects for prioritisation may not always be tangible and data are often lacking or of limited quality, allowing quantification for only a set of pathogens of interest.

An alternative approach to purely quantitative prioritisation is multi-criteria decision analyses (MCDA), a flexible method that enables risk ranking to be based on multiple aspects that compose the risk. These aspects may differ in units, dimensions and scales. MCDA enables prioritisation to be conducted in a systematic and transparent manner by decomposing a complex issue into attributes [12]. These attributes can consist of different ordinal levels. For each topic to be prioritised, in our case FBP, the relevant level per attribute is selected, and the selections are integrated across all attributes to obtain a final ranking score. Topics can subsequently be identified as being of importance based on this score. As such, MCDA can provide a transparent, comparable, and repeatable approach for ranking risks, such as those posed by FBP.

In September 2012, an expert meeting was organised by the Food and Agriculture Organization of the United Nations (FAO) and the World Health Organization (WHO) to use MCDA for determining the relative global importance of 24 FBP, selected from an initial list of 93, with the aim of developing general guidelines for controlling FBP on a global level [4]. *Taenia solium* ranked first in this global exercise, but it was recognised that the ranking is likely to differ among regions and continents. Thus, it was recommended that this type of prioritisation be repeated at regional levels [4]. This recommendation was taken up by COST Action FA1408, A European Network for Foodborne Parasites (Euro-FBP), which organised an expert meeting to prioritise FBP for Europe and regions within using the MCDA approach and original parasite selection from FAO/WHO [4] as starting point.

## Methods

**Figure 1:**
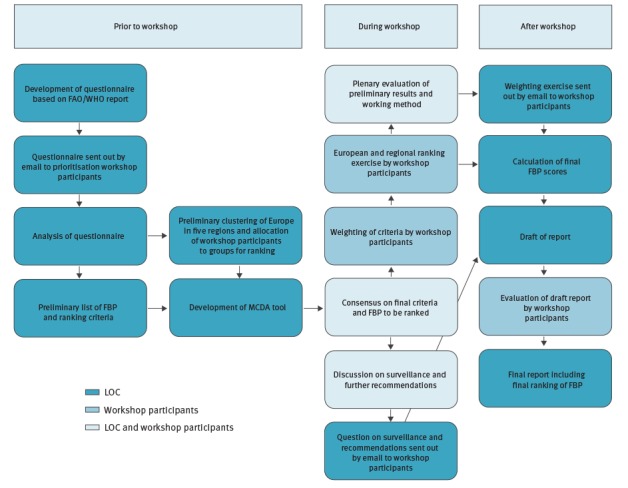
Overview of pre-, intra- and post-workshop activities conducted for the prioritisation of food-borne parasites, 2015 and 2016

FAO: Food and Agriculture Organization of the United Nations; FBP: food-borne parasites; LOC: local organising committee; MCDA: multi-criteria decision analyses; WHO: World Health Organization.

The Euro-FBP MCDA consisted of a workshop as well as pre- and post-workshop activities (Figure 1). The workshop was organised by, and held at, the National Institute for Public Health and Environment (RIVM) in Bilthoven, the Netherlands, from 8–12 February 2016.

### Workshop participants

The prioritisation was conducted as part of Euro-FBP’s work. Several tasks were defined within Euro-FBP, and conducted by dedicated Working Groups (WGs). Participant selection of WGs involved two stages. First, an individual needed to become a member of Euro-FBP by applying through the COST office’s national coordinators via a curriculum vitae and an application form demonstrating knowledge on FBP. Second, an individual needed to apply to join a WG through the Euro-FBP Chair and the WG leader. These processes of self-selection (Euro-FBP application) and selection (WG application) were considered sufficient to assure participation of knowledgeable specialists only. In total, 35 professionals, ranging from early-stage researchers to senior parasitologists, from 25 countries participated in the European ranking and 33 professionals participated in the regional ranking (Table 1). Prior to the workshop, participants were contacted by email and asked to review the parasites and criteria, propose any additions relevant for Europe and inventory any relevant data. These additions were discussed on the first day of the workshop.

**Table 1:** Geographic regions of Europe as considered for European food-borne parasite prioritisation and workshop participant by country, 2016

Geographic region of Europe^a^	Countries included	Participant by country (number of participants)^b^
Northern Europe	Denmark, Finland, Iceland, Norway, Sweden	Denmark (3); Norway (1); Sweden (1)
Western Europe	Austria, Belgium, France, Germany, Ireland, Liechtenstein, Luxembourg, the Netherlands, Switzerland, United Kingdom	Belgium (1); France (1); Germany (1); Ireland (1); the Netherlands (1); Switzerland (1); United Kingdom (1)
Eastern Europe	Czech Republic, Estonia, Hungary, Latvia, Lithuania, Moldova, Poland, Romania, Slovakia	Czech Republic (1); Estonia (1); Hungary (1); Latvia (1); Poland (2); Romania (2); Slovakia (1)
South-Western Europe	Andorra, Italy, Malta, Monaco, Portugal, San Marino, Spain	Italy (1); Spain (1); Tunisia (1)^c^
South-Eastern Europe	Albania, Bosnia and Herzegovina, Bulgaria, Croatia, Cyprus, the former Yugoslav Republic of Macedonia, Greece, Kosovo*, Montenegro, Serbia, Slovenia, Turkey	Bulgaria (1); Croatia (1); the former Yugoslav Republic of Macedonia (2); Greece (2); Serbia (4); Turkey (1)

^a^ Geographic regions were defined as per the European Society of Clinical Microbiology and Infectious Diseases (ESCMID) [18].

^b^ All 35 participants contributed to the European ranking; the participant from Belgium and one participant from Romania did not contribute to the ranking within the different parts of Europe.

^c^ Although Tunisia is not part of Europe, professionals from this country are eligible to participate in COST Actions. The professional input on FBP from Tunisia was considered valuable for this prioritisation.

*This designation is without prejudice to positions on status, and is in line with United Nations Security Council Resolution 1244/99 and the International Court of Justice Opinion on the Kosovo declaration of independence.

### List of parasites to be ranked

The parasites that were included in the FAO/WHO ranking in 2012 were used as the starting point for the European ranking (Table 2).

**Table 2:** Parasites included in the European food-borne parasite prioritisation, 2016

Food-borne parasite
*Angiostrongylus cantonesis* ^a^
Anisakidae
*Ascaris* spp.
*Balantidium coli*
*Cryptosporidium* spp.
*Cyclospora cayetanensis*
*Diphyllobothrium* spp.
*Echinococcus granulosus*
*Echinococcus multilocularis*
*Entamoeba histolytica*
*Fasciola* spp.
*Giardia lamblia*
Heterophyidae and *heterophyidiasis*
Opisthorchiidae
*Paragonimus* spp.
*Sarcocystis* spp.
*Spirometra* spp.
*Taenia saginata*
*Taenia solium*
*Toxocara* spp.
*Toxoplasma gondii*
- *T. gondii*, congenital^a^
- *T. gondii*, acquired^a^
*Trichinella* spp. other than *T. spiralis*
*Trichinella spiralis*
*Trichuris trichiura*
*Trypanosoma cruzi*

^a^ Not considered by FAO/WHO in their multicriteria-based ranking for risk management of food-borne parasites [4].

In order to enable complete comparison with the FAO/WHO ranking results, parasites not expected to be found in Europe, except as imported cases, such as *Paragonimus* spp., Heterophyidae and *Spirometra* spp., were included in the prioritisation. Workshop participants supplemented the list of parasites to be prioritised with *Angiostrongylus cantonensis* because of its potential future emergence in Europe given its presence on the Canary Islands [13] and since then the first autochthonous case was reported in France [14]. In addition, for the purpose of the European list, *T. gondii* was divided into its main clinical entities, congenital and acquired toxoplasmosis, according to transmission route to both allow for distinction based on the difference in transmission routes and comparisons with the global burden of food-borne disease study’s ranking based on DALY [5]. Such differences in transmission routes do not exist for the other FBP considered and comparisons of these FBP with the global ranking can be made without having to divide them into sub-classes.

### Criteria for ranking parasites

The criteria used for the ranking organised by FAO/WHO in 2012 were taken as starting point [4] (Table 3).

**Table 3:** Criterion weights for the FAO/WHO criteria or the Euro-FBP criteria as assessed by direct rating or swing weighting, 2016

Criterion (short-form)	Method			
	Direct rating		Swing weighting	
	FAO/WHO^a^	Euro-FBP	Euro-FPB	Euro-FPB
Number of global/European^b^ food-borne illnesses (C1)	0.22	0.23	0.20	0.18
Geographical distribution (C2)	0.13	0.13	0.13	0.13
Morbidity severity (C345)	0.22	0.23	0.22	0.19
Case–fatality ratio (C6)	0.15	0.15	0.22	0.18
Increasing Illness potential (C7)	0.07	0.10	0.10	0.09
Trade relevance (C8)	0.10	0.09	0.07	0.08
Impact on economically vulnerable communities (C9)	0.10	0.07	0.07	0.08
Introduction probability (C10)^c^	–	–	–	0.07

Euro-FBP: COST Action FA1408, European Network for Foodborne Parasites; FAO: Food and Agriculture Organization of the United Nations; WHO: World Health Organization; –: not assessed.

^a^ Weights assessed and used in the global ranking by FAO/WHO conducted in 2012 [4].

^b^ ‘Global’ in the ranking published by FAO/WHO, ‘European’ in the Euro-FBP ranking.

^c^ Criterion not used in the global ranking by FAO/WHO [4].

The decision rules were identical to those described by FAO/WHO in 2012 with one modification: the values per bin for the criterion ‘number of European food-borne illnesses’ were reduced 10-fold for Europe given the lower population size. The pre-workshop inventory conducted by participants led to the inclusion of one additional criterion: the probability of introduction of a parasite should it not be present in Europe at the time of ranking. At a global level, this criterion was not necessary, but a non-global scale, it was deemed useful to enabling consideration of future developments leading to possible parasite introductions in the various regions. Identification of expected emergence can be informative for preparedness planning. The bins used for scoring this criterion were taken from Havelaar et al. [11] and ranged from < 1%, 1–9%, 10–99% and 100% (the latter bin in case the parasite is already present).

### Determination of criteria weights

In the FAO/WHO ranking, the various criteria were considered not to be of equal importance [4]. Therefore, unequal criteria weights were used in the aggregation of criteria scores into a final prioritisation score (the formula to calculate this score is described in a subsequent section). In the current study, weights for the criteria were also assessed to examine whether their importance at the global level was applicable to the European level according to participants. Two methods, to monitor any method-effect, were used to assess the weights: direct rating (DR), as employed by FAO/WHO in 2012, and swing weighting (SW) [15]. During the workshop, the participants were trained in these procedures and performed an exercise in weighting. The weights that were used for the final ranking were obtained via email after the workshop.

With DR, workshop participants attributed *s_x_* points to each criterion *x* without considering the criteria levels. The sum of *s_x_* over all criteria was 100. The weight for criterion *x*, *w_x,_* was calculated as *s_x_*/100 and averaged across all participants.

With SW, 10 scenarios were scored whereby per scenario, one of the criteria was set at the level of highest importance to public health, while the others were set at the level of least importance, and one additional scenario with all criteria set at the level of least importance. First, the participants ranked the scenarios 1 to *n* from worst to best case based on their opinions regarding the importance of the scenarios with respect to public health (criteria levels were now considered, as opposed to with DR). One hundred points were attributed to the highest-ranking, i.e. most important, scenario and participants decided on the number of points to attribute to the scenario ranking second. A number close to 100 indicated near equal importance to the top-ranked scenario, while lower numbers indicated otherwise. The third-ranked scenario was then attributed points equal to, or lower than, those for the second-ranked scenario based on level of importance, and this procedure continued for each scenario. The final weights were calculated by dividing the score attributed to a criterion by the sum of scores over all criteria, and averaging over all participants. Uncertainty in the weights was obtained by bootstrapping using R software, version 3.3.1 [16]. The dataset with criteria scores per expert/specialist was resampled 100,000 times with replacement, resulting in 100,000 simulated datasets and corresponding criteria weights. The uncertainty distributions thereby obtained for the criteria weights were summarised by their mean and a 95% uncertainty interval, defined as the distribution’s 2.5th and 97.5th percentile.

The difference between unequal and equal weights (i.e. *w_x_* = 0.125) was assessed by analysis of variance (ANOVA) using the normalised weights from the swing weighting for the Euro-FBP criteria. The null hypothesis of equal weights was tested at a 0.05 significance level.

### Computation of final ranking scores

This study focused on three different rankings: a European ranking based on the FAO/WHO methodology and FAO/WHO weights, allowing for comparison with the global ranking; a European ranking based on an additional criterion (10 criteria in total) and using the weights as assessed after the workshop (Euro-FBP weights); and regional rankings for Europe based on the 10 criteria and the Euro-FBP weights.

The ranking score for a parasite for comparison with the global ranking [4], *R_FAO/WHO_*, was calculated as a weighted sum, as done in the FAO/WHO ranking, and averaged over the groups that scored a particular parasite:

$$R_{FAO/WHO}=C_{1}w_{1}+C_{2}w_{2}+\left[C_{3}\left(1-C_{5}\right)+C_{4}C_{5}\right]w_{345}+C_{6}w_{6}+C_{7}w_{7}+C_{8}w_{8}+C_{9}w_{9}$$

where *C_x_* indicates the criterion value for criterion *x* and *w_x_* the corresponding criterion weight. The European ranking score for a parasite including the introduction probability, *R_EU,_*, was computed similarly, but based on different criteria and weights:

$$R_{EU}={C_{1}w}_{1}+C_{2}w_{2}+\left[C_{3}\left(1-C_{5}\right)+C_{4}C_{5}\right]w_{345}+C_{6}w_{6}+C_{7}w_{7}+C_{8}w_{8}+C_{9}w_{9}+I_{int}C_{10}w_{10}$$

where *I_int_* equals 0 if the pathogen is already present in Europe (European ranking) or the region (regional ranking) and 1 otherwise.

The effect on the ranking of unequal weights vs equal weights was examined for the European ranking. To this end, the preference weights were replaced by a weight of 0.125 for all criteria, i.e. all *w_x_* = 0.125 in the above equations. This sensitivity analysis was applied to *R_EU_* only as we considered this outcome most appropriate for the prioritisation in Europe.

### Prioritisation procedure for European ranking

For the European ranking, workshop participants were divided into eight groups, with regional diversity within each group. Group-sizes were four to five participants. Each group was provided with 11 FBP to rank, with each parasite being ranked by at least three different groups. The order of parasites was randomised per group. The choice of the levels per criterion was ideally based on scientific literature, including currently unpublished data that participants were aware of. When empirical evidence lacked, a consensus expert/specialist opinion was reached within groups [17]. After completion, data from all groups were collated in a database and compared. If the choice of levels per criterion per parasite differed by more than one level among the groups (levels are ordinal), these discordances were presented and discussed in a plenary forum with all participants during the workshop. Furthermore, if the difference among groups between the lowest and highest ranking score per parasite would result in ≥ 10 places difference in rank (assessed per parasite, compared with the mean ranking score for other parasites), then the level-choices per group were discussed in the plenary forum as well. Levels were adjusted, without showing the effect on the ranking score, based on consensus, to a maximum difference of one level between groups, to indicate variability in data and/or opinions. Participants were informed about the ranking of parasites based on the new scores on the following day of the workshop, and were asked to identify those parasites with an unexpectedly high or low ranking. Criteria scores for these parasites were discussed again in a plenary session on the last day of the workshop, and selected levels that were considered to be incorrect due to demonstrable data or reasoned argument were adjusted accordingly, without showing the effect on the ranking score.

### Prioritisation procedure for regional ranking

For the regional rankings, the country divisions were based on definitions used by the European Society of Clinical Microbiology and Infectious Diseases. Countries were divided into Western Europe, Northern Europe, Eastern Europe, South-Western Europe, and South-Eastern Europe [18] (Table 1). The Western, Eastern and South-Eastern regions were sub-divided into two groups of participants, with each group scoring the criteria for each parasite independently. Prior to this exercise, all participants were provided with a list of scores for region-independent criteria (i.e. C3, C4, C5 and C6) to speed up the process. Each group scored all parasites, which were presented to groups in a randomised order. Scores from groups that focused on the same region were compared at the end of the workshop for inconsistencies, discussed among the groups and adjusted where deemed appropriate. Rankings for regions that were considered by one group only were reviewed after the workshop by the group participants, with other colleagues where possible, to enable peer checking for these groups as well.

## Results

### Weights assessment

Weights as assessed by the FAO/WHO experts in 2012 and by the participants in the workshop in 2016 differed in decimal points but were quite similar overall in terms of effect on the final ranking score. This is also true for weights obtained by either DR or SW (Table 3). With all approaches, the number of cases of illness and the severity of illness were considered the most important criteria in the ranking, whereas increasing illness potential (C7), trade relevance (C8), and impacts on economically vulnerable communities (C9) were considered to be of lesser importance.

The 95% uncertainty intervals derived by bootstrapping deviated 0.02 units from the average weight for C1, C6, C8 and C9, and 0.01 units for the other criteria. These weights differed significantly from equal weights (p < 0.001). Since the between-group variation in the ranking score was overall larger than the uncertainty, we focused on the variability only in the final rankings.

### European ranking based on FAO/WHO criteria and weights

**Figure 2:**
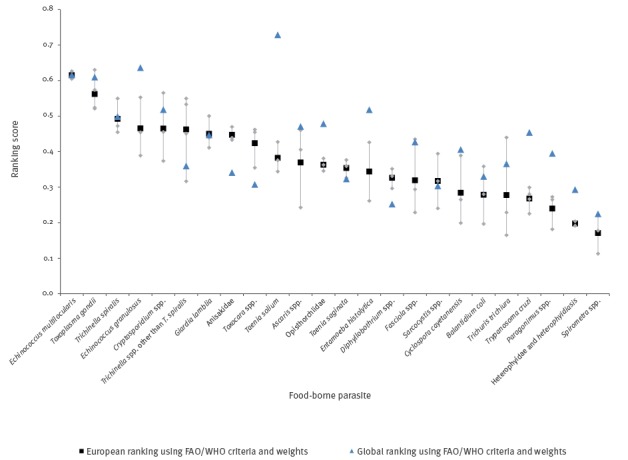
European ranking of food-borne parasites based on the FAO/WHO criteria and weights, and comparison with the FAO/WHO ranking^a^, 2016

FAO: Food and Agriculture Organization of the United Nations; WHO: World Health Organization.

^a^ The FAO/WHO ranking was conducted in 2012 [4].

Figure 2 shows the European ranking based on the FAO/WHO methodology and weights. Most notable was the lower, 10th place rank for *T. solium* for Europe compared with its first place in the global ranking. The parasites ranking second (*E. granulosus*), third (*E. multilocularis*) and fourth (*T. gondii*) at the global level were also among the top four in the European ranking. The biggest difference in rank was observed for *T. cruzi*, ranking 10th globally and 21st in Europe.

The grey diamonds show the scores of the different groups, the grey lines the range in scores.

### European ranking based on Euro-FBP criteria and weights

**Figure 3:**
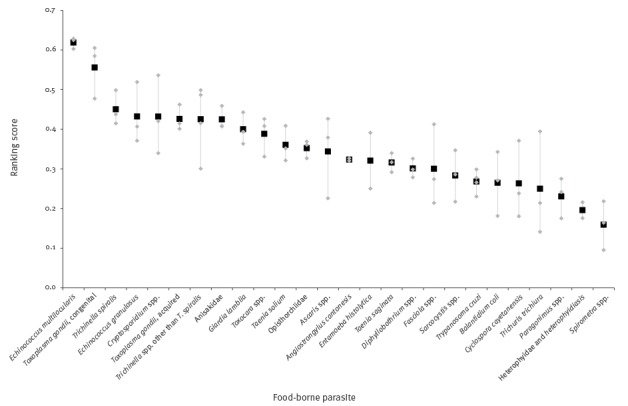
European ranking of food-borne parasites based on the Euro-FBP criteria and weights, 2016

The ranking of parasites using the Euro-FBP criteria and Euro-FBP weights was similar to the ranking based on the FAO/WHO criteria and weights (Figure 3).

The grey diamonds show the scores of the different groups, the grey lines the range in scores.

**Figure 4:**
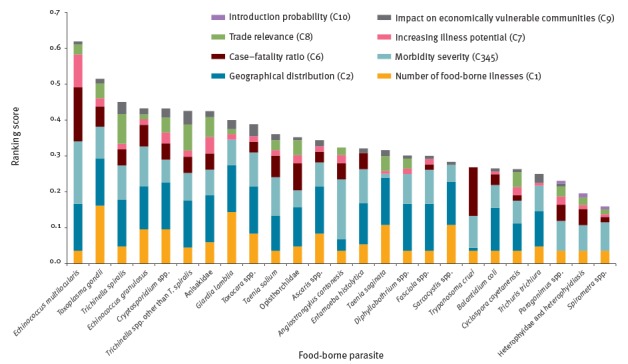
Score breakdown for the European ranking of food-borne parasites based on the Euro-FBP criteria and weights, 2016

This is probably due to the large similarities in weights and the modest impact of the newly-added criterion on the probability of introduction (C10). The parasite that scored as most important in the European ranking was *E. multilocularis*, followed by *T. gondii*. For the former, the criteria ‘geographical distribution’ (C2), ‘morbidity severity’ (C345), and ‘case–fatality ratio’ (C6) contributed to the majority of the ranking score, whereas for *T. gondii* the criteria ‘number of food-borne illness cases’ (C1) and ‘geographical distribution’ (C2) conferred more than 50% of the ranking score (Figure 4).

**Figure 5:**
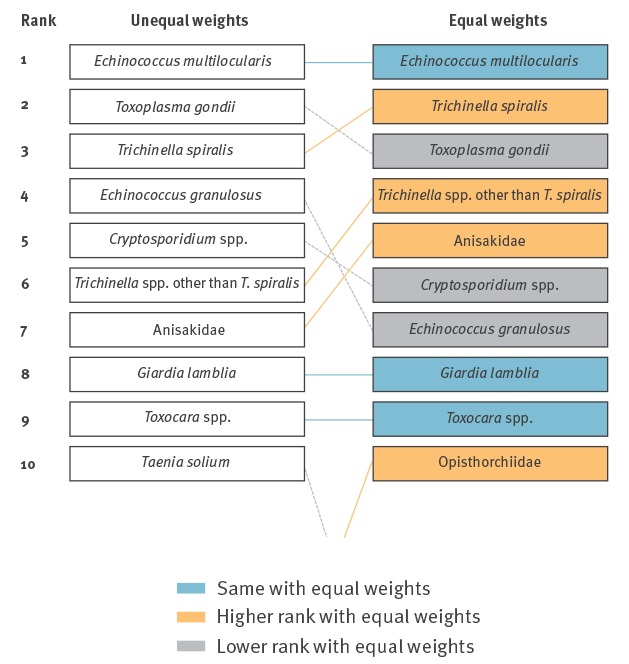
Change in top-10 ranking of food-borne parasites in Europe when replacing the assessed unequal criteria weights with equal weights for ranking based on the Euro-FBP criteria, 2016

Employing equal weights to all criteria left the top-10 largely unaltered compared to the weighted results, with Opisthorchiidae replacing *T. solium* at the 10th place (Figure 5).

### Regional European rankings based on Euro-FBP criteria and weights

**Figure 6:**
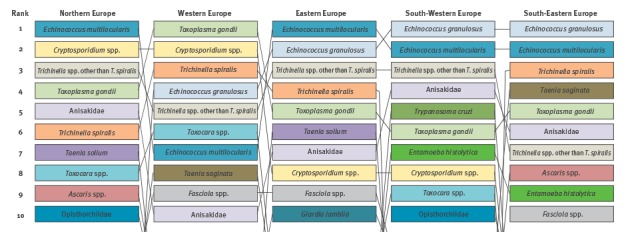
Comparison of the ranking of food-borne parasites in different parts of Europe based on the Euro-FBP criteria, 2016

Differences in priorities were found between different regions of Europe (Figure 6).

The most consistent top-priority FBP in almost all regions was *E. multilocularis*, ranking first or second in all parts except Western Europe where it was ranked seventh. *T. gondii* was considered the top priority in Western Europe, and was ranked third to fifth in all other regions. The rank position of *Cryptosporidium* spp. and *T. spiralis* showed the greatest variability among the top-10 ranked parasites within Europe.

## Discussion

This study acted on the FAO/WHO’s 2012 recommendation to repeat their global ranking of FBP on a regional level. Using the same MCDA approach, as well as an expanded version that included ‘introduction probability’ as an additional criterion, FBP were ranked at the European level and for the different regions within Europe. Overall, *E. multilocularis* and *T. gondii* were considered the most important FBP in terms of public health for Europe as a whole, but regional variability in priorities was found.

Priorities in FBP differed between the global [4] and European level. For example, *Taenia solium* ranked first in the global ranking, but 10th in the European ranking. The main reason for this is that *T. solium* is practically absent in Europe whereas it remains common in many Asian, South American and African countries [19]. In the European ranking, *E. multilocularis* and *T. gondii* were among the priority FBP. For *E. multilocularis*, the cause of alveolar echinococcosis in humans, there is a lack of autochthonous cases of illness in some parts of Europe, e.g. Northern Europe, while it is endemic in some countries in Western Europe where it ranked seventh, making this outcome unexpected [20]. However, a low number of illnesses was scored for most FBP considered in the prioritisation, with the notable exception of *T. gondii* and *Giardia*, making this criterion not a main determinant of the prioritisation. In hindsight, using more discriminative levels might have been of added benefit. The relatively high severity of morbidity and the case–fatality ratio mainly drove the ranking score for *E. multilocularis*, supplemented with a predicted increase in spread of this parasite within Europe, and thus the threat of potential future illness. For *T. gondii*, the dominant criterion driving the ranking score were the number of cases of illness and geographical distribution. The priority of *T. gondii* has previously been suggested based on other metrics, including DALY [5,21] and economic impact [22].

Overall, the MCDA-procedure yields a transparent and repeatable outcome while simultaneously striving to maximise objectivity and fact-based reasoning. Nevertheless, for some criteria-levels that were chosen, it was not possible for each FBP to base these on scientific data or measurements, as FBP-specific data were not available. In such cases, the selected criteria-levels were based on professional opinions after within-group discussions. Ideally, professional opinions from each country in Europe would have been obtained to further limit bias in the prioritisation, but in the current study, the professionals originated from 25 of 49 countries considered by this study to be in Europe. This incomplete coverage resulted from the selection procedure we used and is often the case in MCDA, whether by geography, discipline, normative background, etc. [12]. The implications of potential regional bias on the current prioritisations are impossible to assess. Although FBP professionals may be rarer or difficult to reach in certain countries, a more active search for professionals per country or an open call for expressions of interest to participate could have resulted in a more complete coverage. Such an approach is therefore recommended for future prioritisations.

Another limitation related to the use of professional opinion when objective data are absent, is the introduction of subjectivity in the results. By scoring each FBP at least three times, i.e. by three different groups, and providing a plenary forum to discuss discordant opinions, i.e. those criteria where there was more than one level difference, we aimed to attain the most appropriate and least subjective choice given the available knowledge. Misclassification could, however, have occurred, and the choices made should, ideally, be updated when relevant data become available.

The MCDA provided a ranked list of FBP in Europe. Although parasites are attributed a ranking number, the absolute value of the ranking score was very similar for certain parasites, e.g. *E. granulosus*, *Cryptosporidium* and *Trichinella* spp. other than *T. spiralis*. Furthermore, the range of ranking scores among groups varied considerably for some FBP, which could, apart from regional differences in importance, be due to a lack of available data. Hence, results from this ranking should be seen as a general indication of parasites that are expected to be of greater importance or lesser importance for public health. Nonetheless, no better, more feasible alternative to our approach is available to provide a structured, transparent and fair comparison of FBP, or infectious diseases in general, for prioritisation. Indeed, a publication by the European Food Safety Authority also notes that when empirical evidence is limited, which occurs rather often, expert judgement may be necessary instead [17].

The numerical value of the criteria weights can be an important determinant in the ranking. As such, the methodology selected for determining weights, as well as the selection of the panel providing the weights, can influence the ranking. To reduce methodological bias, the participants obtained hands-on training during the workshop and provided independent score assessments via email after the workshop. Our comparative approach in assessing the weights shows that the weights are generally consistent across two professional panels (FAO/WHO expert group and Euro-FBP participants; only two professionals participated in both studies) and the two methodologies used (direct rating and swing weighting). Furthermore, our sensitivity analysis, employing equal weights for all criteria, showed that the top-10 priority FBP remained unchanged with the exception of that in the 10th place (data not shown). As such, we expect our choices regarding the weights to have had a negligible influence on the prioritisation.

In conclusion, *E. multilocularis* and *T. gondii* ranked highest at the European level. Hence, FBP that are considered of greatest importance to Europe differ from those that are considered of greatest importance globally, which were *T. solium* and *E. granulosus*. This finding reflects important differences in the distribution and public health risks of FBP. The results of this ranking exercise may be used to inform public health decision makers about where resources for FBP surveillance systems might be most usefully directed and also to provide input into developing a research agenda on FBP in Europe.

## Notes

**Euro-FBP workshop participants:** Hafidh Akkari (ENMV, Tunisia), Teofilia Banu (IDAH, Romania), Rabia Cakir Koc (YTU, Turkey), Rachel Chalmers (Wales NHS, UK), Carmen Michaela Cretu (UMFCD, Romania), Gunita Deksne (Bior, Latvia), Olgica Djurkovic-Djakovic (UoB, Serbia), Pierre Dorny (ITM, Belgium), Emilia Dvoroznakova (SAS, Slovakia), Heidi L Enemark (NVI, Norway), Cedric Gerard (Nestlé, Switzerland), Maria Angeles Gomez Morales (ISS, Italy), Maja Jurhar-Pavlova (UKiM, the former Yugoslav Republic of Macedonia), Christian Kapel (UCPH, Denmark), Titia Kortbeek (RIVM, the Netherlands), Istvan Kucsera (ANTSZ, Hungary), Brian Lassen (EMU, Estonia) Jelena Petrovic (NIV, Serbia), Katharina Riehn (HAW Hamburg, Germany), David Rodriguez-Lazaro (UBU, Spain), Miroslaw Rozycki (NVRI, Poland), Michal Slany (VRI, Czech Republic), Jovana Stefanovska (UKiM, the former Yugoslav Republic of Macedonia), Mario Sviben (HZJZ, Croatia), Krzysztof Stojecki (NVRI, Poland), Smaragda Sotiraki (NAGREF, Greece), Chiara Trevisan (ITM, Belgium), Karin Troell (SVA, Sweden), Alexandra Uzelac (UoB, Serbia), Isabelle Vallee (ANSES, France), Apostolos Vantarakis (UPATRAS, Greece), Sasa Vasilev (INEP, Serbia), Kamenna Vutova (MU-Sofia, Bulgaria), Theo de Waal (UCD, Ireland)
